# An Investigation into Rumen Fungal and Protozoal Diversity in Three Rumen Fractions, during High-Fiber or Grain-Induced Sub-Acute Ruminal Acidosis Conditions, with or without Active Dry Yeast Supplementation

**DOI:** 10.3389/fmicb.2017.01943

**Published:** 2017-10-10

**Authors:** Suzanne L. Ishaq, Ousama AlZahal, Nicola Walker, Brian McBride

**Affiliations:** ^1^Ishaq Informatics, LLC, Bozeman, MT, United States; ^2^AB Vista, Marlborough, Wiltshire, United Kingdom; ^3^Animal Biosciences, University of Guelph, Guelph, ON, Canada

**Keywords:** SARA, rumen pH, fungal ITS, protozoal 18S, mothur, dairy cattle

## Abstract

Sub-acute ruminal acidosis (SARA) is a gastrointestinal functional disorder in livestock characterized by low rumen pH, which reduces rumen function, microbial diversity, host performance, and host immune function. Dietary management is used to prevent SARA, often with yeast supplementation as a pH buffer. Almost nothing is known about the effect of SARA or yeast supplementation on ruminal protozoal and fungal diversity, despite their roles in fiber degradation. Dairy cows were switched from a high-fiber to high-grain diet abruptly to induce SARA, with and without active dry yeast (ADY, *Saccharomyces cerevisiae*) supplementation, and sampled from the rumen fluid, solids, and epimural fractions to determine microbial diversity using the protozoal 18S rRNA and the fungal ITS1 genes via Illumina MiSeq sequencing. Diet-induced SARA dramatically increased the number and abundance of rare fungal taxa, even in fluid fractions where total reads were very low, and reduced protozoal diversity. SARA selected for more lactic-acid utilizing taxa, and fewer fiber-degrading taxa. ADY treatment increased fungal richness (OTUs) but not diversity (Inverse Simpson, Shannon), but increased protozoal richness and diversity in some fractions. ADY treatment itself significantly (*P* < 0.05) affected the abundance of numerous fungal genera as seen in the high-fiber diet: *Lewia, Neocallimastix*, and *Phoma* were increased, while *Alternaria, Candida Orpinomyces*, and *Piromyces* spp. were decreased. Likewise, for protozoa, ADY itself increased *Isotricha intestinalis* but decreased *Entodinium furca* spp. Multivariate analyses showed diet type was most significant in driving diversity, followed by yeast treatment, for AMOVA, ANOSIM, and weighted UniFrac. Diet, ADY, and location were all significant factors for fungi (PERMANOVA, *P* = 0.0001, *P* = 0.0452, *P* = 0.0068, Monte Carlo correction, respectively, and location was a significant factor (*P* = 0.001, Monte Carlo correction) for protozoa. Diet-induced SARA shifts diversity of rumen fungi and protozoa and selects against fiber-degrading species. Supplementation with ADY mitigated this reduction in protozoa, presumptively by triggering microbial diversity shifts (as seen even in the high-fiber diet) that resulted in pH stabilization. ADY did not recover the initial community structure that was seen in pre-SARA conditions.

## Introduction

Sub-acute ruminal acidosis (SARA) is a well-recognized gastrointestinal functional disorder in ruminant livestock, characterized by periods of low rumen pH which are often driven by a sudden switch to a highly-fermentable, starch-based diet. The physiological effects of a decreased rumen pH, as well as the associated decrease in feed intake and downstream gastrointestinal dysfunction (i.e., diarrhea) of SARA cause subsequent reductions in rumen function, microbial diversity, host performance, and host immune function (Khafipour et al., 2009; Hook et al., 2011; Petri et al., 2013; McCann et al., 2016; Sato, 2016). Additionally, acidosis can lead to other systemic health problems, such as liver abscesses or inflammation (including laminitis) (reviewed in Plaizier et al., 2008). Moreover, changes to the environmental and functional rumen ecosystems (liquid-associated, solid/particle-associated, and host-epithelium associated) drive changes to host gene expression and epithelial function, as well as shifts in microbial diversity and functionality (Steele et al., 2011; Petri et al., 2013; McCann et al., 2016; AlZahal et al., 2017).

Dietary management is the most widely-used technique for preventing the onset of SARA in cattle (Stone, 2004). The effects of yeast supplementation on preventing or treating SARA, as well as on bacterial diversity, have been previously characterized (Khafipour et al., 2009; Petri et al., 2013; AlZahal et al., 2014, 2017; Uyeno et al., 2015; McCann et al., 2016). Yet almost nothing is known about its effect on ruminal protozoal and fungal diversity, despite their roles in fiber degradation (Williams and Withers, 1993; Lee et al., 2000; Krause et al., 2003; Sun et al., 2006; Belanche et al., 2012a,b).

Rumen microorganisms are highly susceptible to changes in rumen pH driven by dietary carbohydrate profiles, which has been well-characterized for bacteria (ex. Henderson et al., 2015). High-fiber diets favor rumen fungal diversity (Belanche et al., 2012a), as well as cellulolytic protozoal genera such as *Polyplastron, Eudiplodinium*, and *Epidinium* (Michałowski et al., 1991; Béra-Maillet et al., 2005; Kittelmann and Janssen, 2011). High-starch diets, on the other hand, favor the protozoa *Entodinium* (Dehority and Odenyo, 2003), although it should be noted that the Dehority and Odenyo results were likely differential by species, as only some *Entodinium* (i.e., *E. caudatum*) have been shown to be amylolytic. High starch diets have been shown to have no effect (Hristov et al., 2012; Boots et al., 2013), to reduce total abundance (Belanche et al., 2012a), to reduce diversity within three common genera (Denman et al., 2008), and to reduce diversity in sequenced libraries (Kumar et al., 2015; Tapio et al., 2017).

While fungi are negatively affected by a decrease in pH, they may be positively affected by the reduction in bacteria with which they are often in competition for nutrients (Møller et al., 1999). For example, *Neocallimastix frontalis*, a cellulolytic fungus, was inhibited by the cellulolytic bacterium *Ruminococcus flavefaciens* (Bernalier et al., 1993). *In vitro* studies found *Saccharomyces cerevisiae* yeast reduced bacterial protease activity (Chaucheyras-Durand et al., 2005), and could clear *Escherichia coli* from rumen fluid (Chaucheyras-Durand et al., 2010). However, bacterial-fungal interactions can be rather positive, and can even include cross-domain production of growth-promoters (reviewed in Tarkka et al., 2009). *In vivo* studies under SARA conditions showed treatment with *S. cerevisiae* active-dry yeast (ADY) improved rumen pH (Bach et al., 2007; Thrune et al., 2009; AlZahal et al., 2014), as well as adherent bacteria (ex. *Fibrobacter succinogenes*) abundance, and total microbial cellulolytic mRNA abundance (AlZahal et al., 2014, 2017).

Fungal abundance and cellulolytic potential were found to increase in the presence of hydrogen-utilizing species, such as methanogenic archaea (Joblin et al., 1990; Marvin-Sikkema et al., 1990), presumably due to the pH-modulating effect. Many species of rumen protozoa and methanogenic archaea are known to interact symbiotically (Vogels et al., 1980; Sharp, 1998; Ohene-Adjei et al., 2007), but there exists an antagonism between fungi and protozoa. For example, many protozoa produce hydrogen during fiber digestion (Krumholz et al., 1983), there is competition for fiber substrates, some protozoal enzymes have been shown to degrade fungal cell walls (reviewed in Gruninger et al., 2014), while others consume fungal spores (Hsu et al., 1991; Morgavi et al., 1994). Given the complexity of biological interactions, as well as chemical reactions in the rumen, it may be that dietary changes and ADY intervention cause indirect changes to rumen community structure, which have implications for rumen function recovery.

This study sought to (1) identify protozoal and fungal diversity in cows fed a high-fiber diet in epimural, fluid, and solid-associated fractions, (2) determine the changes in protozoal and fungal diversity in the rumen of cows with diet-induced SARA, (3) determine the effect of ADY supplementation on rumen diversity under a high-fiber diet, and (4) determine whether ADY treatment could rescue protozoal and fungal diversity if it was negatively affected by SARA. It was hypothesized that the shift in diet substrate to a high-grain diet, and the resulting acidification of rumen fluid, would shift the diversity of both microorganism types, and that treatment with ADY would rescue rumen alpha-diversity.

## Methods

### Animals, feeding and treatments, and rumen sampling

This protocol has been detailed previously (AlZahal et al., 2014, 2017). All experimental procedures were approved by the University of Guelph Animal Care Committee (animal utilization protocol 12R050), in accordance with the Canadian Council on Animal Care (CCAC, 1993). In summary, 16 multiparous, second-lactation Holstein dairy cows (166 ± 30 DIM), ~650–750 kg, with rumen cannula, were randomly assigned to either a control group (*n* = 8) or a treatment group (*n* = 8). The treatment group were given a yeast supplement (*S. cerevisiae*; AB Vista, Marlborough, UK; 8 × 10^10^ cfu/head per day) which was applied as a top dressing, and which was prepared weekly by mixing 4 g of ADY (2 × 10^10^ cfu/g of DM) with 250 g of ground dry corn (AlZahal et al., 2014). Either the ADY or the ground corn carrier only (control) were administered to cows daily for the entire 10 week study.

Prior to the trial, all cows had been maintained on TMR and were naïve to the yeast supplement. For the first 6 weeks, all cows received a high forage (HF) diet (77:23, forage:concentrate; CP = 14.3, NDF = 45.0, NFC = 31.5, % of DM) (AlZahal et al., 2014) to create optimal rumen conditions. All cows were abruptly transitioned during a 24 h period in week 7 to a high grain (HG) diet (49:51, forage:concentrate; CP = 16.4, NDF = 28.2, NFC = 45.2, % of DM) (AlZahal et al., 2014) to induce SARA. During the 24 h transition period, cows were only given 50% of the grain ration; the following day cows received the full ration and remained on the HG until the end of week 10. The four groups (high-fiber control, HFC; high-fiber + yeast, HFY; high-grain control, HGC; and high-grain + yeast, HGY) allowed for multiple group comparisons to elucidate the effects of diet, yeast supplementation, and diet + yeast supplementation on rumen fungal and protozoal communities.

Feed intake, milk yields, and pH were recorded daily on an individual basis and were previously reported (AlZahal et al., 2014). Rumen samples for DNA-based analysis were collected as detailed previously (AlZahal et al., 2017). Briefly, cows were sampled at wk5 (HF) and wk10 (HG) at 1,600 h. Whole contents were sampled via direct grab through the cannula from the ventral sac of the rumen, with fluid and particle-associated fractions separated by cheesecloth filtration and stored independently at a 1:1 with 100% ethanol until bacterial genomic DNA isolation (AlZahal et al., 2014). To obtain epimural samples, the rumen was partially evacuated, and a small section halfway into the ventral sac was washed with cold PBS to remove adherent particles. The washed area was swabbed with a sterile toothbrush, and the toothbrush was vortexed in a 50 ml tube with 25 ml PBS to remove microorganisms. The epimural samples were then fixed with 25 ml of 100% ethanol (AlZahal et al., 2017).

### DNA extraction and sequencing

Nucleic acids were extracted and prepared for Illumina MiSeq (Illumina, San Diego, CA) at the University of Guelph sequencing facility as previously described (AlZahal et al., 2014, 2017) using the repeated bead-beating method (Yu and Morrison, 2004). Protozoa were amplified using previous protocols (Ishaq and Wright, 2014; Ishaq et al., 2015) that utilized the primers P-SSU-316F (5′-GCTTTCGWTGGTAGTGTATT-3′) (Sylvester et al., 2004) and GIC758R (5′-CAACTGTCTCTATKAAYCG-3′) (Ishaq and Wright, 2014) which target the V3–V4 region of the 18S rRNA gene and signature regions 3–4. The Internal Transcribed Spacer 1 region (ITS1) of fungi was amplified using the primers ITS5 (5′-GGAAGTAAAAGTCGTAACAAGG-3′) and ITS2 (5′-GCTGCGTTCTTCATCGATGC-3′) (White et al., 1990). Sequencing library prep was performed according to previously published protocols using the KAPA HiFi HotStart PCR kit (KAPA Biosystems, Wilmington, MA). PCR product was cleaned and normalized with a SequalPrep Normalization Kit (Invitrogen, ThermoFisher Scientific, US) (AlZahal et al., 2016, 2017), and pooled at equimolar concentrations. All DNA isolation, library preparation, and sequencing took place shortly after the animal trial in 2014. Sequences are available from NCBI under BioProject accession number PRJNA386328, for both fungi (*n* = 95 samples) and protozoal (*n* = 89) community datasets.

### Sequence and statistical analysis

Fungal ITS and protozoal 18S datasets were processed independently of one another: each had barcodes and primers removed with default parameters by the sequencing facility, and were processed using mothur ver. 1.38 (Schloss et al., 2009). For fungal data, paired-end sequences were separated from jointly-run 16S sequences using trim.seqs in mothur to parse by primer, and then sequences were culled if they contained ambiguous bases, were shorter than 90 (Zimmerman and Vitousek, 2012) or longer than 487 bases, or which did not align or classify to the Findley fungal ITS database (Findley et al., 2013) which had been *de novo* aligned in-house using MUSCLE (Edgar, 2004). An in-house ruby script was used to truncate sequences at the reverse primer or at homopolymers after 8 bases (Luo et al., 2012; Ishaq et al., 2017). Significance between group means of taxonomic relative abundance is listed in Supplementary Tables 1, 2, and all standard error means were <0.07 for fungi and <0.05 for protozoa (data not shown). Paired-end protozoal sequences were assembled into contigs using PANDAseq (Masella et al., 2012), and culled if they contained ambiguous bases or homopolymers >8 bases, were shorter than 500 or longer than 550 bases, or which did not align and classify to a rumen ciliate protozoal 18S database (Ishaq and Wright, 2014).

For statistical analysis, protozoa were subsampled (normalized) to 5,000 sequences/sample, and fungi were subsampled to 500 sequences/sample due to low reads/sample (Supplemental Table 3; Smith et al., 2014). As a comparison, fungi were also subsampled at 1,000 reads/sample, which did not dramatically alter clustering or statistical comparisons; however, it more severely reduced the number of samples which could be used for statistical comparison, thus the normalization was set at 500 sequences. Both datasets were clustered using the nearest neighbor method, protozoal at a 4% species-level cutoff (Ishaq and Wright, 2014) and fungi at a 3% species-level cutoff (Blaalid et al., 2013). Diversity was calculated using the mothur-integrated versions of CHAO (Chao and Shen, 2003), ACE (Chao and Shen, 2010), Good's Coverage (Etsy, 1986), Inverse Simpson (Simpson, 1949), and Shannon Diversity (Shannon and Weaver, 1949), with significant differences (*P* < 0.05) calculated using Student's *T*-test for pairwise comparisons. Linear discriminant analysis (Segata et al., 2011) was used to determine discriminatory OTUs by treatment group, with significance at *P* < 0.05 using Wilcoxon rank test. Bray-Curtis Dissimilarity was calculated using mothur and used to compare samples, upon which analysis of molecular variance (AMOVA), analysis of similarity (ANOSIM), and UniFrac (Lozupone and Knight, 2005) were performed using the mothur-integrated versions. Treatment effects were also measured using PERMANOVA with a mixed-effects model in PRIMER ver 6. (Clarke, 2006), following square-root transformation and Bray-Curtis Dissimilarity. Non-Metric Multidimensional Scaling Plots (NMDS) based off Bray-Curtis Dissimilarity were visualized in R (R Core Team, 2015) using ggplot2. A heatmap of significant Pearson's correlations between treatment parameters and OTU abundance was created in R using the corrplot package, which generated correlations and tested significance.

## Results

**Figure 1:**
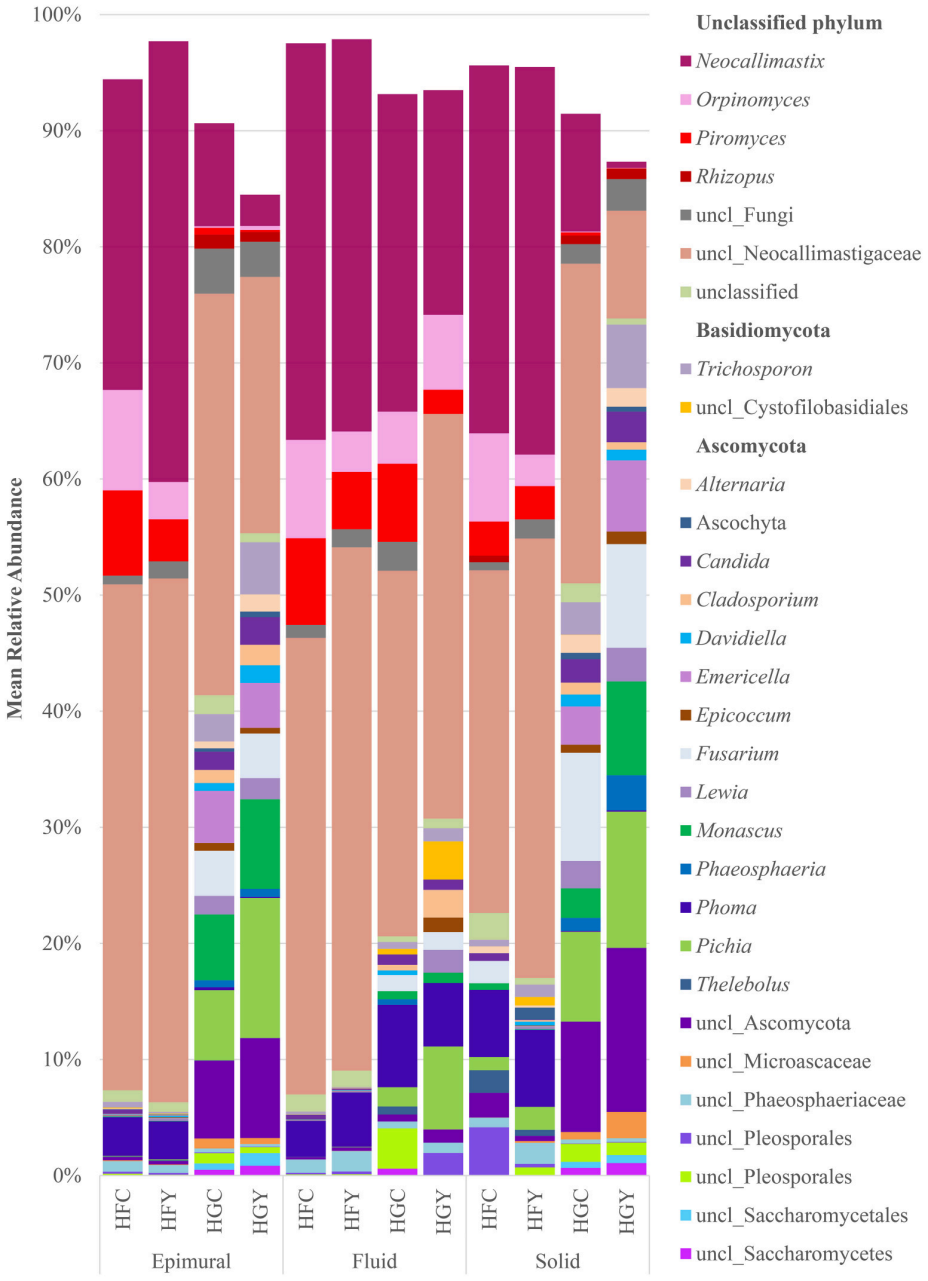
Relative abundance of rumen fungi genera for cows receiving a high fiber (HF) or high grain (HG) diet, with (Y) or without (C) yeast supplementation. Treatments include high-fiber control (HFC), high-fiber yeast (HFY), high-grain control (HGC), and high-grain yeast (HGY).

A total of 262 fungal genera were identified, with 103 having a significant difference between at least two treatment groups (Supplemental Table 1). Taxonomic diversity was significantly different when comparing controls by diet (HFC and HGC) and ADY treatments by diet (HFY and HGY) in all three sample locations for fungi (Figure 1, Supplemental Table 1), but less so when comparing control to ADY treatment within either the HF or HG diet (C and Y). Taxonomic diversity was also different between HFC and HGY, indicating that ADY supplementation did not recover the initial fungal community. The taxonomic diversity of fungi showed a dramatic increase in the proportion of rare taxa (<1% abundance) from a HF to a HG diet (Figure 1, shown as blank). When comparing control to yeast treatment in the HF diet, *Lewia* and *Neocallimastix* spp. relative abundance were notably increased with yeast treatment in multiple fractions, while *Phoma* was increased in fluid. *Alternaria, Candida, Orpinomyces*, and *Piromyces* spp. relative abundance were decreased in HFY. *Saccharomyces* all classified as *S. cerevisiae*, though to multiple strains (data not shown), but were not found in >1% mean relative abundance in any treatment group or significantly more abundant in any group.

**Figure 2:**
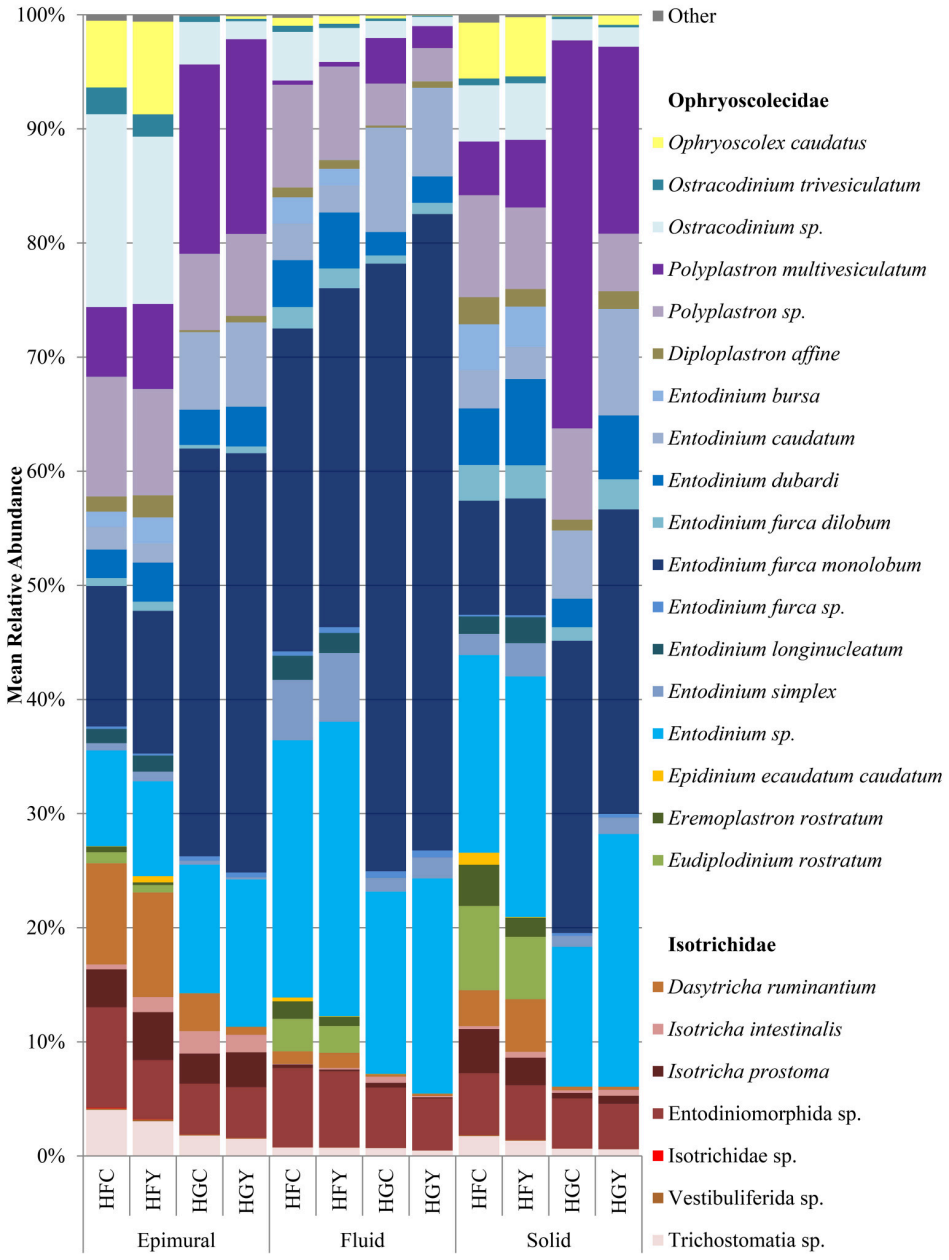
Relative abundance of rumen protozoal species for cows receiving a high fiber (HF) or high grain (HG) diet, with (Y) or without (C) yeast supplementation. Treatments include high-fiber control (HFC), high-fiber yeast (HFY), high-grain control (HGC), and high-grain yeast (HGY).

A total of 44 protozoal species were identified, with 38 having a significant difference between at least two treatment groups (Supplemental Table 2). The relative abundances of the protozoa *Entodinium furca monolobum, Entodinium caudatum*, and *Polyplastron multivesiculatum* were significantly increased in all sample locations in the HG diet over the HF diet (Figure 2, Supplemental Table 2). Likewise, relative abundances of *Ophryoscolex caudatus, Ostracodinium trivesiculatum, Epidinium ecaudatum, Eremoplastron rostratum, Eudiplodinium rostratum*, and *Dasytricha ruminantium* were significantly decreased in the HG diet. When comparing control to yeast treatment in the HF diet, *Isotricha intestinalis* and other *Isotricha* species' abundances were increased, while *E. furca* spp. were decreased. When comparing control to yeast treatment in the HG diet, *P. multivesiculatum* and *Entodinium* spp. were increased, while *E. rostratum, Eremoplastron* spp., *Ostracodinium gracile*, and other *Ostracodinium* spp. relative abundance were decreased. Taxonomic diversity was also different between HFC and HGY, indicating that ADY supplementation did not recover the initial protozoal community.

**Figure 3:**
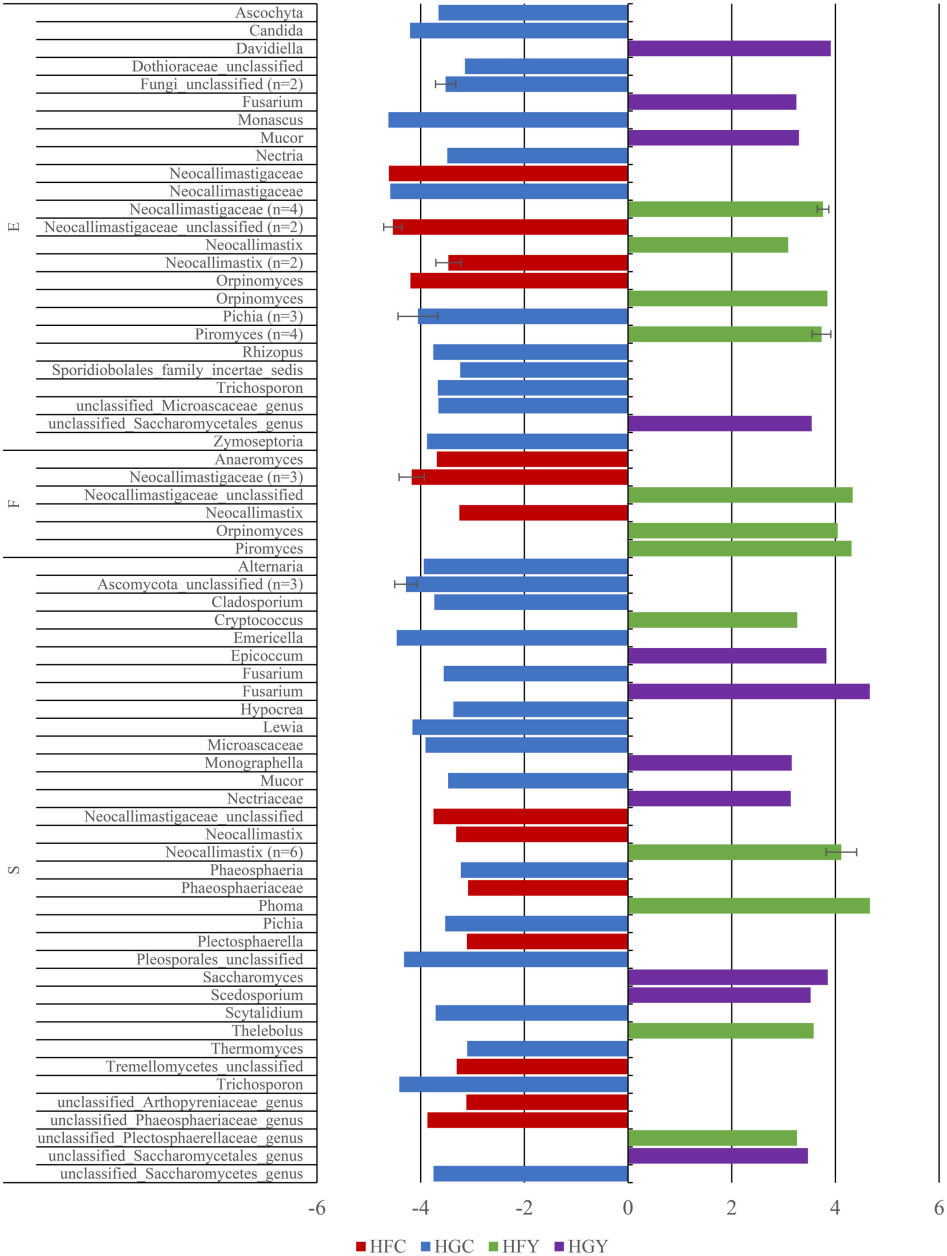
Linear Discriminant Analysis of significant fungal OTUs in the epimural (E), fluid (F), and solid (S) fractions for cows receiving two dietary treatments with or without yeast supplementation under SARA conditions. Error bars represent standard deviation for OTUs with multiple LDA values. Treatments include high-fiber control (HFC), high-fiber yeast (HFY), high-grain control (HGC), and high-grain yeast (HGY).

**Figure 4:**
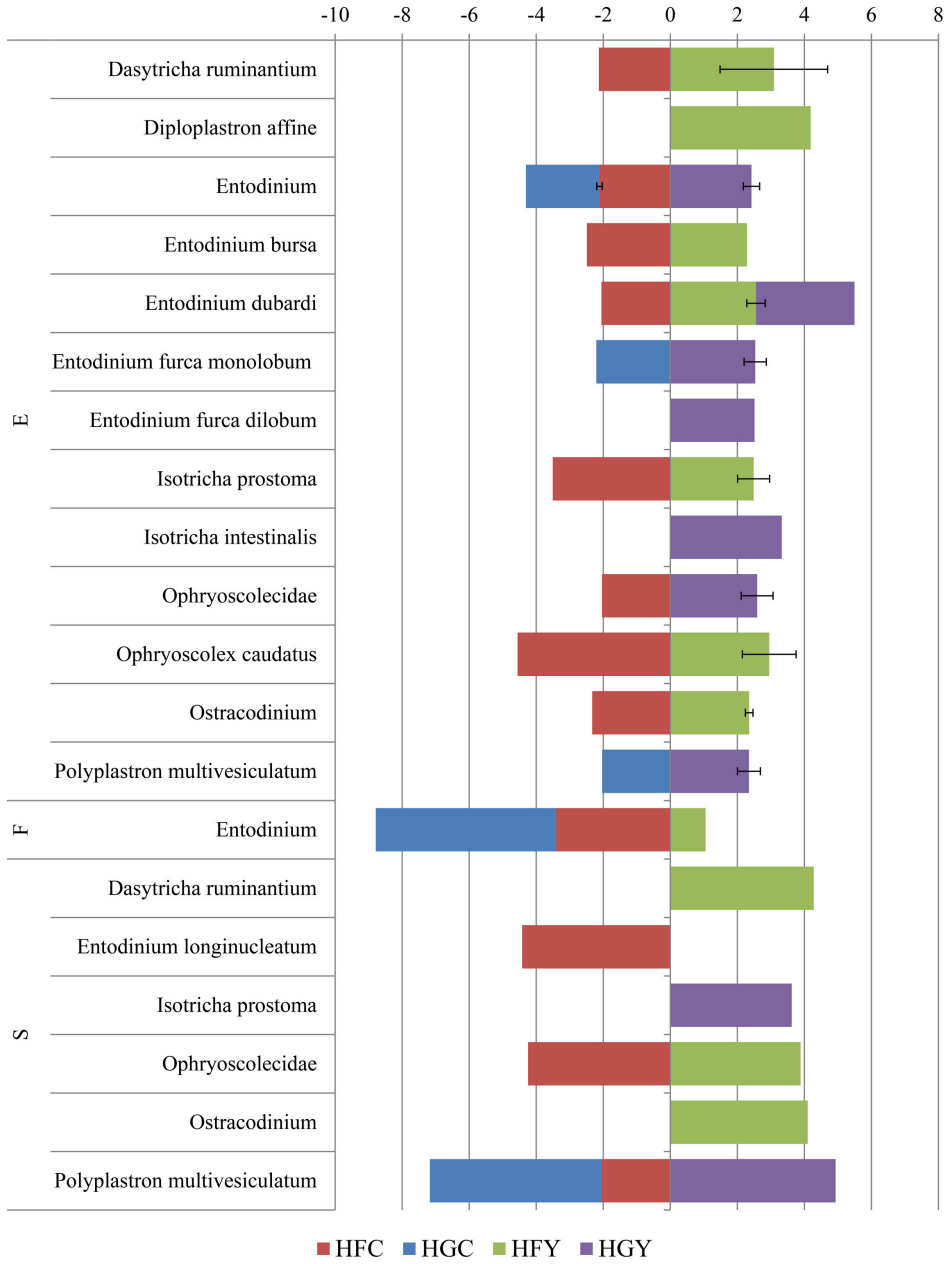
Linear Discriminant Analysis of significant protozoal OTUs in the epimural (E), fluid (F), and solid (S) fractions for cows receiving two dietary treatments with or without yeast supplementation under SARA conditions. Error bars represent standard deviation for OTUs with multiple LDA values. Treatments include high-fiber control (HFC), high-fiber yeast (HFY), high-grain control (HGC), and high-grain yeast (HGY).

Linear discriminant analysis was used to determine significant OTUs by treatment group for fungi (Figure 3) and protozoa (Figure 4). Diet was delineated by 59 fungal and 7 protozoal OTUs, and location by 35 fungal and 45 OTUs. ADY was delineated by 5 fungal OTUs; one genus *Orpinomyces* and four family Neocallimastigaceae, and 1 protozoal OTU: genus *Entodinium*.

Observed fungal OTUs were significantly higher in solid fractions of HGY than HGC, and solid fractions of HFY trended (*P* < 0.06) toward being significantly higher than HFC (Table 1). ACE was higher in solid HFC than solid HGC for fungi. Inverse Simpson and Shannon-Weiner Diversity were higher in epimural and solid fractions of HFY than HFC, and HGC had higher diversity than HFC in the epimural and solid fractions. HG diets saw no fungal samples which had enough read coverage to be statistically compared in the fluid fraction. Protozoal samples showed greater differences in observed OTUs, CHAO, and ACE by sample location and treatment group, with epimural samples showing greater diversity than fluid or solid-associated samples (Table 1). HFC had more OTUs than HGC in the solid fraction; however, the HGY epimural fractions showed higher OTUs as compared to HFY or HGC. Inverse Simpson and Shannon Diversity showed multiple significant interactions between treatments: yeast increased diversity in both diets, and HF diet fractions were more diverse than HG fractions.

**Table 1:** Statistical diversity for rumen fungi and protozoa for cows receiving two dietary treatments with or without yeast supplementation under SARA conditions.

**#samples**	**CHAO**	**ACE**	**OTUs**	**Good's coverage**	**Inverse simpson**	**Shannon-Weiner**
**FUNGAL ITS**						
**HFC**						
Epimural (7)	89 ± 14	154 ± 67	53 ± 7	95%±1^a^	12 ± 4^a^	2.88 ± 0.2^ac^
Fluid (8)	124 ± 94	110 ± 35	51 ± 8	96%±0	13 ± 3	2.97 ± 0.2
Solid (5)	97 ± 20	129 ± 27^a^	54 ± 8^T^	95%±1^T^	11 ± 3^b^	2.91 ± 0.3^b^
**HFY**						
Epimural (8)	111 ± 31	136 ± 40	58 ± 7	95%±1	15 ± 3	3.12 ± 0.2^c^
Fluid (8)	108 ± 45	113 ± 54	54 ± 7	96%±1	14 ± 3	3.08 ± 0.2
Solid (3)	102 ± 20	167 ± 66	62 ± 9^T^	94%±1	11 ± 4	2.94 ± 0.2
**HGC**						
Epimural (6)	103 ± 29	108 ± 38	68 ± 8	94%±2^a^	19 ± 4^a^	3.38 ± 0.1^a^
Fluid (0)	–	–	–	–	–	–
Solid (8)	91 ± 28	92 ± 29^a^	61 ± 6^a^	96%±1^T^	18 ± 4^b^	3.29 ± 0.2^b^
**HGY**						
Epimural (7)	102 ± 18	129 ± 24	64 ± 12	95%±1	19 ± 9	3.14 ± 0.5
Fluid (0)	–	–	–	–	–	–
Solid (8)	111 ± 33	131 ± 56	62 ± 5^a^	95%±1	15 ± 5	3.14 ± 0.3
**PROTOZOAL 18S**						
**HFC**						
Epimural (6)	876 ± 207	1773 ± 646	271 ± 38	93%±2	3 ± 1^c^	2.13 ± 0.2^d^
Fluid (8)	28 ± 64	65 ± 173	15 ± 28	99.5%±1	2 ± 1^d^	0.63 ± 0.5^e^
Solid (8)	9 ± 3^a^	6 ± 5^a^	9 ± 3^a^	100%±0.1	3 ± 2^a^	1.39 ± 0.5^a^
**HFY**						
Epimural (7)	992 ± 245	2070 ± 647	298 ± 50^b^	92%±2^a^	5 ± 2^c^	2.48 ± 0.4^d^
Fluid (7)	283 ± 484	511 ± 889	115 ± 189	96%±7	5 ± 5^d^	1.77 ± 1.4^b, e^
Solid (7)	9 ± 2^b^	4 ± 5	9 ± 2^c^	100%±0.0	4 ± 2^b^	1.53 ± 0.4^c^
**HGC**						
Epimural (8)	961 ± 206	2134 ± 981	282 ± 56^d^	93%±2.4^b^	2 ± 1^d^	1.67 ± 0.7^f^
Fluid (7)	5 ± 2	3 ± 3	5 ± 2	100%±0.1	2 ± 1	0.50 ± 0.4
Solid (8)	6 ± 2^a^	2 ± 3^a^	5 ± 2^a^	100%±0.3	2 ± 1^a^	0.85 ± 0.5^a^
**HGY**						
Epimural (7)	1000 ± 146	1773 ± 583	381 ± 47^b, d^	90%±1.7^a, b^	5 ± 2^d^	2.88 ± 0.5^f^
Fluid (7)	4 ± 2	1 ± 2	4 ± 2	100%±0.0	2 ± 1	0.57 ± 0.5^b^
Solid (7)	6 ± 2^b^	4 ± 3	6 ± 2^c^	100%±0.0	2 ± 1^b^	0.88 ± 0.5^c^

*Superscripts represent significant (P < 0.05) differences by Student's T-Test, for fungal and protozoal diversity separately, compared by row for each measure. T indicates a trending P-value; 0.05 < T < 0.06*.

*Treatments include high-fiber control (HFC), high-fiber yeast (HFY), high-grain control (HGC), and high-grain yeast (HGY). Error is presented as standard deviation*.

**Figure 5:**
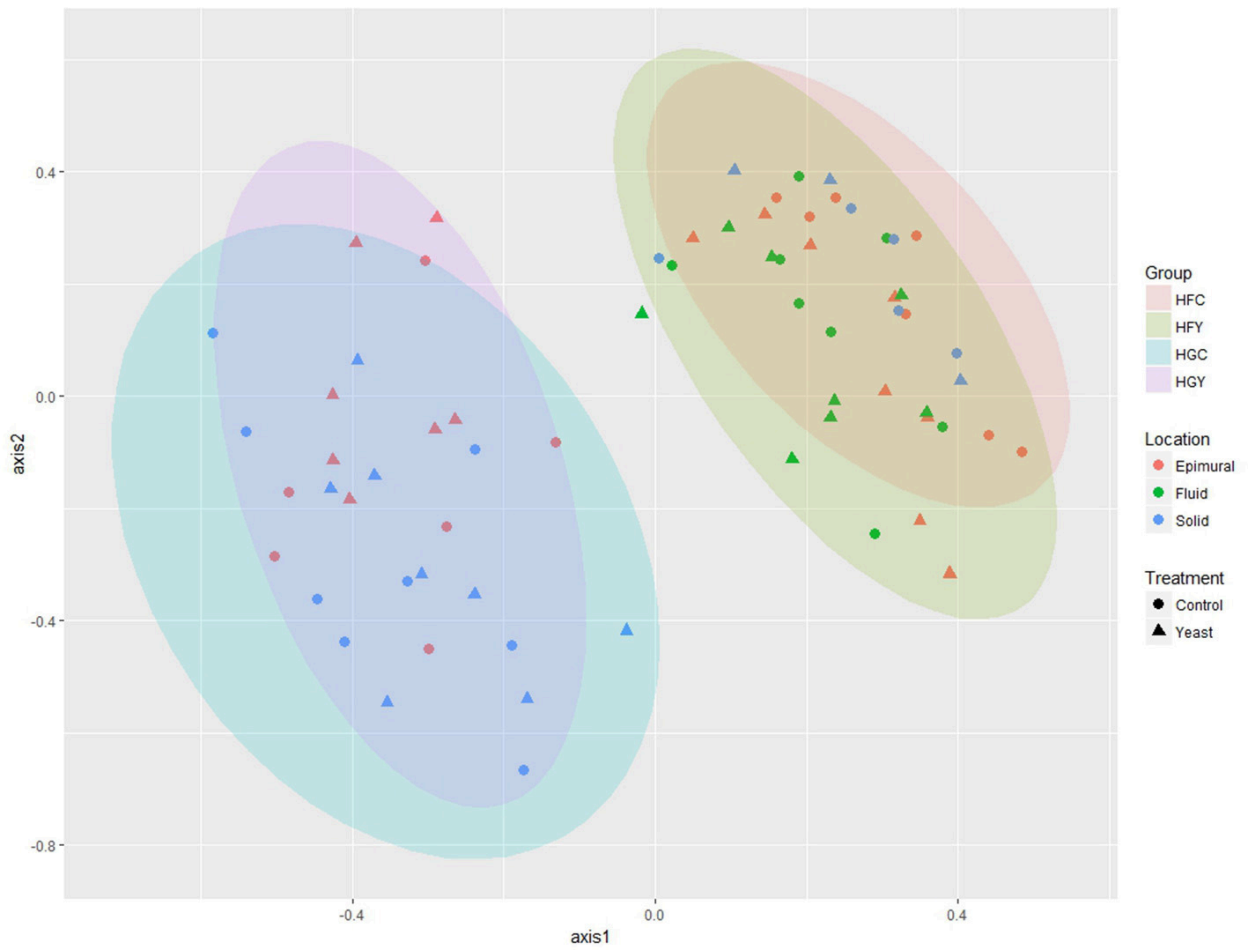
Non-metric Multidimensional Scaling (nMDS) plot for rumen fungi from cows receiving two dietary treatments with or without yeast supplementation under SARA conditions. Lowest stress = 0.13, *R*^2^ = 0.93.

**Figure 6:**
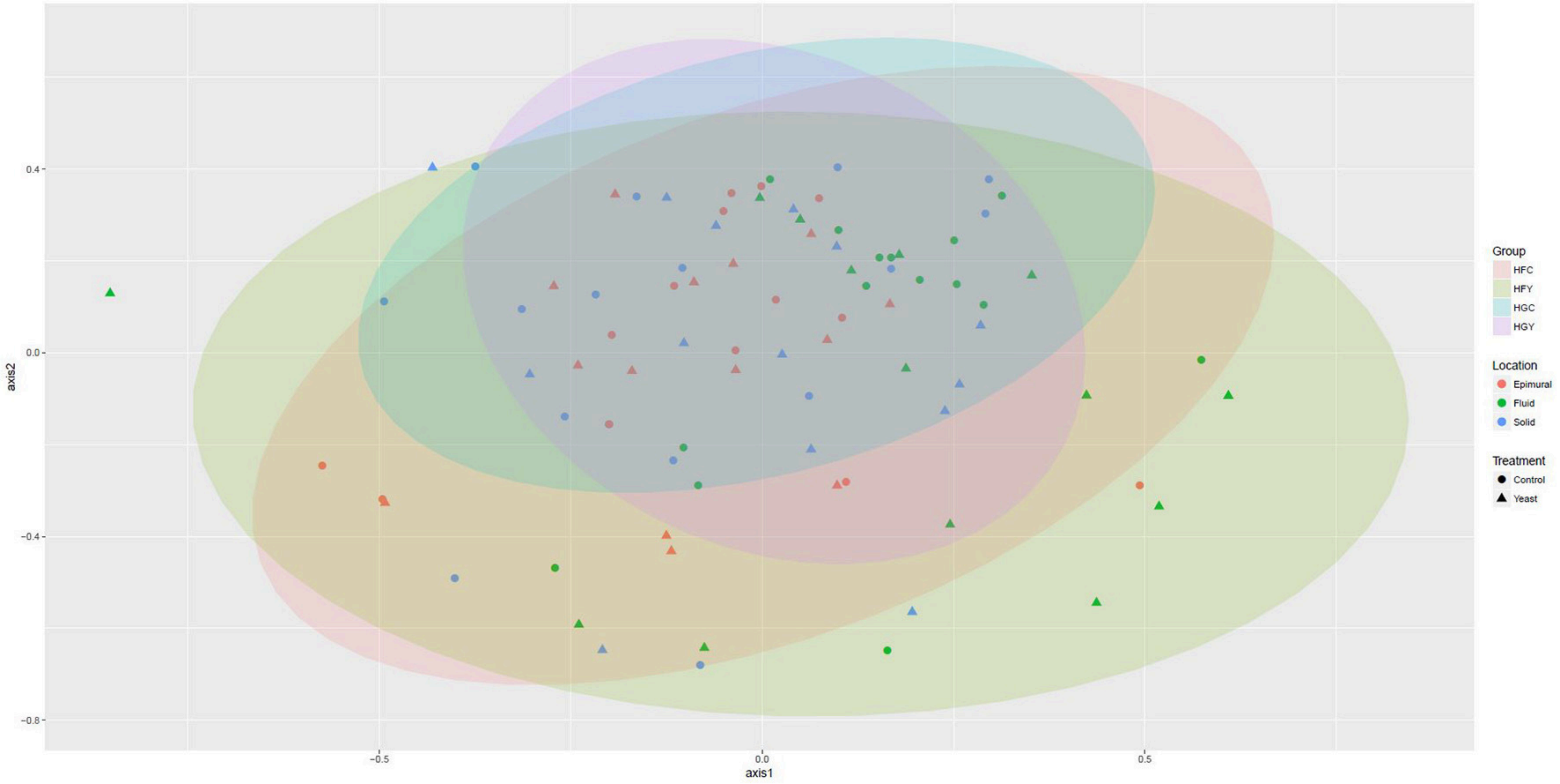
Non-metric Multidimensional Scaling (nMDS) plot for rumen protozoa from cows receiving two dietary treatments with or without yeast supplementation under SARA conditions. Lowest stress = 0.19, *R*^2^ = 0.80.

Multivariate analyses showed diet type was very significant in driving diversity, followed by ADY treatment, for AMOVA, ANOSIM, and weighted UniFrac (Table 2). The interactions between treatment and diet were often location-specific, with significant differences seen largely in epimural fractions and occasionally in solid fractions. This was visually confirmed using NMDS for both fungi (Figure 5) and protozoa (Figure 6). PERMANOVA indicated that diet (*P* = 0.0001, MC) ADY (*P* = 0.0452, MC), and location (*P* = 0.0068, MC) were all significant factors for fungi. However, only location was a significant factor (*P* = 0.001, Monte Carlo correction) for protozoa using PERMANOVA repeated measures. When comparing HFC to HGY to determine whether ADY treatment rescued diversity, fungal communities were still distinct, while protozoal populations were not significantly different (Table 2). However, protozoal populations were not significantly different for many comparisons, thus overlap between HFC and HGY likely reflects that the treatment effects on protozoa were low rather than a rescuing of diversity with ADY.

**Table 2:** Comparison of treatments by AMOVA, ANOSIM, and UniFrac, for rumen fungi and protozoa for cows receiving two dietary treatments with or without yeast supplementation under SARA conditions.

	**Fungal ITS**					**Protozoal 18S**				
	**AMOVA**	**ANOSIM**		**Weighted UniFrac**		**AMOVA**	**ANOSIM**		**Weighted UniFrac**	
	***P***	***R***	***P***	***W***	***P***	***P***	***R***	***P***	***W***	***P***
Location	^**^	0.13	^*^	0.65	^**^	^*^	0.08	^**^	0.87	^**^
Epimural × Fluid	^**^	0.05	ns	0.65	^**^	^**^	0.10	^*^	0.99	^**^
Epimural × Solid	T^a^	0.06	ns	0.55	^**^	^*^	0.08	^*^	1	^**^
Fluid × Solid	^**^	0.28	^**^	0.77	^**^	^*^	0.07	^*^	0.61	^**^
HF × HG	^**^	0.93	^**^	1	^**^	^**^	0.10	^**^	0.65	^**^
C × Y	ns	0.01	ns	0.48	^**^	ns	0.00	ns	0.61	^**^
Treatment	^**^	0.51	^**^	0.83	^*^	^**^	0.15	^**^	0.87	^**^
**HFC** × **HGC**										
Epimural	^**^	0.91	^**^	1	^**^	ns	0.40	^*^	1	^**^
Fluid	n/a	n/a	n/a	n/a	n/a	ns	0.00	ns	0.65	^**^
Solid	^**^	0.95	^**^	1	^**^	ns	0.11	ns	0.74	^**^
**HFY** × **HGY**										
Epimural	^**^	0.82	^**^	1	^**^	ns	0.31	^*^	1	ns
Fluid	n/a	n/a	n/a	n/a	n/a	ns	0.19	^*^	0.5	^**^
Solid	T^a^	0.85	T1	1	^**^	ns	0.00	ns	0.85	^**^
**HFC** × **HFY**										
Epimural	ns	0.03	ns	0.61	^*^	ns	−1.8	ns	0.96	^**^
Fluid	ns	0.01	ns	0.55	^*^	ns	0.03	ns	0.66	^**^
Solid	ns	0.00	ns	0.79	^*^	ns	0.00	ns	0.65	^**^
**HGC** × **HGY**										
Epimural	ns	0.02	ns	0.74	^**^	ns	0.31	^*^	0.95	^*^
Fluid	n/a	n/a	n/a	n/a	n/a	ns	0.00	ns	0.72	^**^
Solid	ns	0.00	ns	0.63	^**^	ns	0.02	ns	0.67	^**^
**HFC** × **HGY**										
Epimural	^**^	0.84	^**^	1	^**^	^*^	0.32	T^a^	0.95	^*^
Fluid	n/a	n/a	n/a	n/a	n/a	ns	0.00	ns	0.53	^**^
Solid	^**^	0.84	^**^	1	^**^	ns	0.00	ns	0.74	^**^

a *Values were significant only before Bonferroni correction*.

*Diets include high fiber (HF) or high grain (HG), locations include Epimural (E), fluid (F), or solid (S), and treatments include yeast (Y) or Control (C). Significance is determined as P < 0.05*,

* *P < 0.001*,

** *P > 0.05 (ns), or not enough comparisons to make (n/a). Significance was adjusted by Bonferroni where appropriate*.

**Figure 7:**
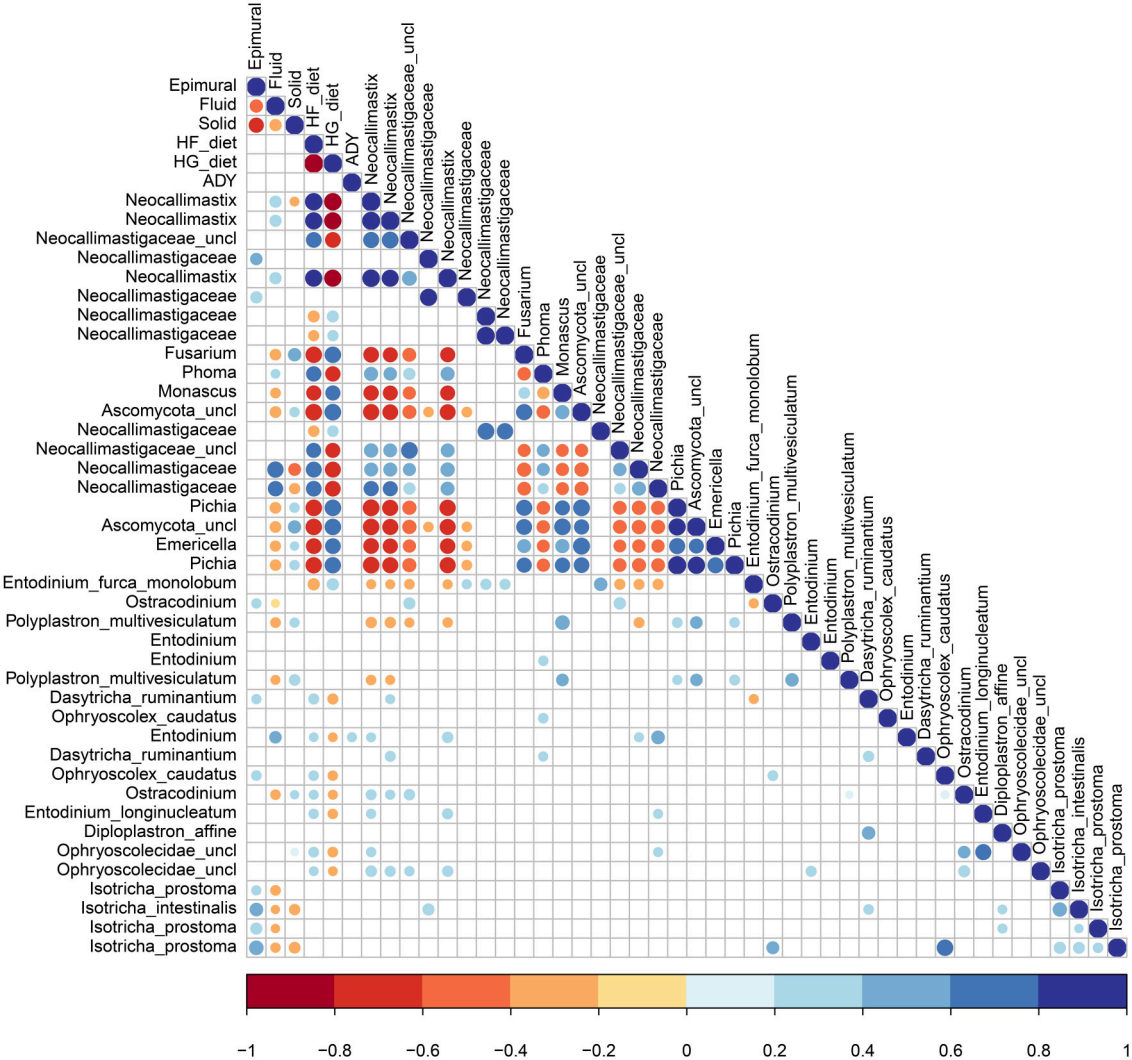
Significant Pearson's correlations between diet, active dry yeast supplementation, and rumen location, with the top 20 fungal and protozoa OTUs.

Pearson's correlations indicate significant correlations (*P* < 0.05) among fungi, among protozoa, between kingdoms, and for both diet and ADY supplementation (Figure 7). Fungi in the Neocallimastigaceae family were positively correlated with HF and the fluid fraction, while the genera *Emericella, Fusarium, Monascus*, and *Pichia* were positively correlated with HG and the solid fraction. None of the top 20 fungal OTUs, and only one protozoa *Entodinium* sp., was positively correlated with ADY. Protozoa were correlated with a HF diet, with the exception of *E. furca monolobum*. All the top OTUs identified as protozoal *Isotricha* spp., were positively associated with the epimural fraction, along with a few other species. Fungal-protozoal correlations were largely positive. *E. furca monolobum* had several negative fungal correlations, but this was likely due to its correlation with HG. *Polyplastron multivesiculatum*; however, had several negative fungal correlations which were independent of diet.

## Discussion

In the present study, rumen protozoal and fungal diversity was reported in dairy cows fed a high-fiber diet in epimural, fluid, and solid-associated fractions to describe baseline populations under normal rumen conditions (objective 1). Protozoal sequences were identified in epimural fractions, contrary to a previous study which used a different variable region of the 18S rRNA gene (Shin et al., 2004). Following diet-induced SARA, the diversity of protozoal was reduced, especially in fractions associated with the rumen wall, and the fiber-degrading species were notably altered (objective 2). However, in the present study, diet-induced SARA increased fungal diversity, which is contrary to some previous findings which showed no effect (Hristov et al., 2012; Boots et al., 2013), a reduction in total abundance (Belanche et al., 2012a), a reduction of diversity within three common genera (Denman et al., 2008), and a reduced diversity in sequenced libraries (Kumar et al., 2015; Tapio et al., 2017). This disparity may be a function of setting a biologically inappropriate minimum sequence length cutoff during quality assurance steps, as some fungal ITS sequences are 100–150 bases, ex. *Pichia*, which would otherwise be removed. *Pichia* and *Candida* both contain species which utilize lactic-acid (Mendes de Almeida et al., 2012; Sirisan et al., 2013), and both of which were increased on a high-grain diet in the current study. Likewise, entodiniomorphid protozoa consume lactate (Newbold et al., 1987), thus acidosis does not affect all species similarly.

Grain is generally considered to be a source of fungal spores for livestock, and a number of species have been identified in feed (reviewed in Dicostanzo and Murphy, 2012). While the grain feed was not tested for fungal diversity, *Alternaria* and *Mucor* spp. were both increased post-feeding the HG diet in the present study and have been previously identified in grain (Abe et al., 2015; Lee et al., 2015). In addition to changing the profile of the carbohydrates available in the rumen, switching to a HG diet reduced the pH of the rumen, as previously discussed (AlZahal et al., 2014, 2017), both of which select differential diversity. Diet-induced SARA can cause damage to the rumen epithelium (Steele et al., 2011) and increase the expression of host genes responsible for rumen epithelial barrier function (McCann et al., 2016). Any changes to the structure and function of the rumen epithelium, including those triggered by SARA, may negatively impact the diversity and density of rumen fungi living there. Fungi have the slowest life cycles of rumen microorganisms (24–32 h) (Theodorou et al., 1996; Hobson and Fonty, 1997), and association with the rumen epithelium may help cells avoid wash-out. Fungi (Warner, 1966; Orpin, 1975; Gruninger et al., 2014) and protozoa (Hook et al., 2012; Williams and Coleman, 2012) are known to associate with rumen epithelial cells until chemotaxis draws them into the liquid and solid fractions. In the present study, the greatest changes to diversity and community occurred in epithelial fractions. Any epithelial damage accrued during SARA may then have larger consequences for the recovery of fungal and protozoal diversity and functionality.

Cellulase enzyme activity requires acid catalysis, and as such cellulase activity most often occurs extracellularly in the rumen, is sensitive to local pH, and works best in a slightly acidic environment (pH 6–7) (Weimer, 1993; Russell and Wilson, 1996; Sung et al., 2007). Yet, many cellulolytic microorganisms are not acid tolerant, and the maintenance of a neutral or basic intracellular pH in the context of an acidic extracellular pH can cause some acidic volatile fatty acids to disperse into cells and accumulate to toxic levels as intracellular anions (Russell and Diez-Gonzalez, 1998). Once rumen pH is below 6.0, the extent and duration of the lowered pH will differentially affect the ability of cellulolytic bacteria to attach to fiber particles (Roger et al., 1990; Mouriño et al., 2001; Sung et al., 2007). This window in functionality during acidosis events may account for why diversity of fungi and protozoa was not always significantly changed in solid fractions in the current study, as pH was less delineating between pre- and post-SARA groups than expected because individual pH variation was high (AlZahal et al., 2014).

Under normal rumen conditions and HF diet, the daily addition of ADY modified rumen communities (objective 3). Supplementation with ADY mitigated the reduction in protozoal diversity caused by diet or pH (objective 4), consistent with previous studies on bacteria (AlZahal et al., 2017). The cellulolytic fungi *Neocallimastix*, and the protozoa *I. intestinalis* were all increased by ADY supplementation, even as the cellulolytic fungi *Orpinomyces* and protozoa *E. furca* spp. were decreased. A meta-analysis of *S. cerevisiae* supplementation indicated that it would increase protozoal growth (Desnoyers et al., 2009). Isotrichids have been shown to associate with plant particles due to chemotaxis toward a variety of sugars (Orpin and Letcher, 1978; Diaz et al., 2014a,b), thus their increase in the present study with ADY treatment may result from the associated improvement in fiber digestion and availability of sugars (AlZahal et al., 2014).

In the present study, the reduction in protozoal diversity could be attributed to the change in pH or the change in substrate as grain and concentrate diets often reduce microbial diversity (Wu et al., 2011; Belanche et al., 2012a; Li et al., 2013; Fernandes et al., 2014; Kumar et al., 2015). Differentiating between the effects of the availability of different feed substrates and the acid-production potential of feeds (Kim et al., 2012) on rumen microbial diversity is challenging, especially as different feed substrates or formulations can cause varied amount of saliva production, which can buffer rumen fluid pH. *In vitro* investigation using fermentation chambers showed pH was a larger driver of fermentative ability than substrate; low pH reduced microbial fiber digestion, nitrogen circulation, and volatile fatty acid production, especially acetate and butyrate (Calsamiglia et al., 2007). *S. cerevisiae* not only buffers pH in this (AlZahal et al., 2014) and previous studies (Bach et al., 2007; Thrune et al., 2009), but alters the redox potential of rumen fluid, and with its ability to survive in the rumen allows for a more continuous control of rumen pH, it has an advantage over chemical pH buffering with sodium bicarbonate (Marden et al., 2008).

Previous work has shown indirect competition between fungi and protozoa for fiber and in dealing with hydrogen byproducts (Krumholz et al., 1983), which is often mitigated by associated methanogens (Joblin et al., 1990; Marvin-Sikkema et al., 1990). Protozoa also appear to directly compete with fungi through enzymatic destruction and predation (Hsu et al., 1991; Gruninger et al., 2014), although a meta-analysis has suggested that rumen defaunation more often causes a reduction in cellulolytic microorganisms, including fungi (Newbold et al., 2015). In the present study, fungal-protozoal correlations were largely positive.

As relatively few studies examine fungal-protozoal interactions in the rumen, it is difficult to differentiate between dietary effects and biotic interactions. For example, here, *E. furca monolobum* was negatively correlated with fungi in the Neocallimastigaceae family. *E. furca monolobum* and *Neocallimastix* are cellulolytic and have been associated with methanogens seeking hydrogen (Regensbogenova et al., 2004; Wei et al., 2016), potentially they may be competing for fiber substrate or hydrogentrophs in the rumen. On the other hand, *E. furca monolobum* also had a negative correlation with the HFD that was positively correlated with those fibrolytic species. *Polyplastron multivesiculatum*; however, had several negative fungal correlations which were independent of diet.

Moreover, there can be predatory competition between rumen protozoal populations, especially from *P. multivesiculatum* toward *Entodinium, Epidinium*, and *Eudiplodinium* spp., which dominate Type B rumen populations and are often found in domestic livestock (Eadie, 1967; Coleman et al., 1972; Towne et al., 1988a). *Polyplastron*, along with *Ophyroscolex* and *Metadinium*, dominate Type A rumen populations which are common in wild ruminants (Towne et al., 1988a,b), and may represent a “wild-type community.” Type A will out-compete Type B when added to naïve Type B (Eadie, 1967; Coleman et al., 1972). Previous studies have also reported mixed A/B populations in ruminants (Towne et al., 1988a,b), indicating a potential for stasis in competition at the species' level, as well as Type O (Coleman, 1979), consisting only of *Entodinium, Isotricha*, and *Dasytricha* which are more acid-tolerant (Lyle et al., 1981; Dennis et al., 1983). Sheep have also been shown to change from Type O to other types following diet changes (Kittelmann et al., 2016). In the present study, cows on both diets and treatments hosted a Type A/B population, despite a change in diet and rumen pH.

This study provides an interesting consideration into the effect on less abundant, yet functionally-critical rumen taxa, namely fungi and protozoa, under conditions of diet change, SARA, and supplementation with an ADY. However, a great deal of additional work is needed to elucidate interactions between microbial taxa in the rumen under normal and dysbiotic conditions. Moreover, the definition of a healthy microbiome has yet to be determined in ruminants, particularly where fungi and protozoa are concerned, suffice that more diversity is widely regarded as healthier. While ADY recovered total diversity in some populations in the present study, and did improve the abundance of some fibrolytic taxa; however, it did not rescue the pre-SARA community. Based on previous results using ADY in these particular cows, ADY was shown to improve cattle health (AlZahal et al., 2014) and fibrolytic bacterial abundance (AlZahal et al., 2017).

## Author contributions

SI performed sequencing data analysis and interpretation, and wrote the manuscript. OA performed previous analyses related to and used in this study, consulted on the data analysis, and edited the manuscript. NW provided technical assistance in this and previous portions of the study, and edited the manuscript. BM conceived study design and provided resources, and edited the manuscript.

### Conflict of interest statement

The authors declare that the research was conducted in the absence of any commercial or financial relationships that could be construed as a potential conflict of interest.
