# Supplementary material for: An Investigation into Rumen Fungal and Protozoal Diversity in Three Rumen Fractions, during High-Fiber or Grain-Induced Sub-Acute Ruminal Acidosis Conditions, with or without Active Dry Yeast Supplementation
Source: Front Microbiol. 2017 Oct 10;8:1943. doi: 10.3389/fmicb.2017.01943 (PMC5641310; doi:10.3389/fmicb.2017.01943)
Supplement: Supplementary file 1 [file DataSheet1.docx]

**Supplemental Table 1. Significantly different fungal genera between treatment groups.** Significance is determined as P < 0.05 = * and P < 0.01 ** by Student’s T-Test. Treatments include high-fiber control (HFC), high-fiber yeast (HFY), high-grain control (HGC), and high-grain yeast (HGY). Standard error was < 0.07 for all treatment groups and taxa.

|  | **Epimural** | | | |  | | | | **Fluid** | | | | |  | | | | **Solid** | | | |
| --- | --- | --- | --- | --- | --- | --- | --- | --- | --- | --- | --- | --- | --- | --- | --- | --- | --- | --- | --- | --- | --- |
| **taxon** | **HFC-HFY** | **HGC-HGY** | **HFC-HGC** | **HFY-HGY** | | **HFC-HGY** |  | **HFC-HFY** | | **HGC-HGY** | **HFC-HGC** | **HFY-HGY** | **HFC-HGY** | |  | **HFC-HFY** | **HGC-HGY** | | **HFC-HGC** | **HFY-HGY** | **HFC-HGY** |
| *Alternaria* | ns | * | ** | ** | | ** |  | ns | | ns | ** | ns | ** | |  | ns | ns | | ns | ** | ns |
| *Anaeromyces* | ns | ns | ** | * | | ** |  | ns | | ns | ** | * | ** | |  | ns | ns | | ns | ** | ns |
| *Articulospora* | ns | ns | ** | ns | | ** |  | ns | | ns | ns | ns | ns | |  | ns | ns | | ns | ns | ns |
| *Ascochyta* | ns | ns | ns | * | | * |  | ns | | ns | * | * | * | |  | ns | ns | | ns | ns | * |
| *Aureobasidium* | ns | ns | * | ** | | * |  | ns | | ns | ns | ns | ns | |  | ns | ns | | * | ns | ns |
| *Bensingtonia* | ns | ns | ns | ns | | ns |  | ns | | ns | ns | ns | ns | |  | * | ns | | ns | * | ns |
| *Bulleromyces* | ns | ns | * | ns | | ns |  | ns | | ns | ns | * | ns | |  | ns | ns | | ** | ns | * |
| *Calycina* | ns | ns | ns | * | | ns |  | ns | | ns | ns | ns | ns | |  | ns | ns | | ns | ns | ns |
| *Candida* | ns | ns | * | ** | | * |  | ns | | ns | ns | ns | ns | |  | ns | ns | | ns | ** | ns |
| *Chalara* | ns | ns | * | ns | | ns |  | ns | | ns | ns | ns | ns | |  | * | ns | | ns | * | ns |
| *Cladosporium* | ns | ns | ** | ** | | ** |  | ns | | ns | ns | ns | ns | |  | * | ns | | ** | ** | ** |
| *Cryptococcus* | ns | ns | ns | ns | | ns |  | ns | | ns | ** | ** | ** | |  | * | ns | | ns | ns | * |
| *Cyllamyces* | ns | ns | ns | ns | | ns |  | ns | | ns | ns | ** | ns | |  | ns | ns | | ns | ns | ns |
| *Cystofilobasidium* | ns | ns | ns | ns | | ns |  | ns | | ns | ** | * | ** | |  | * | ns | | ns | ns | ns |
| *Davidiella* | * | ** | ** | ** | | ** |  | ns | | ns | ns | * | ** | |  | ns | ns | | ** | * | ** |
| *Debaryomyces* | ns | ns | * | ns | | ns |  | ns | | ns | * | ns | * | |  | ns | ns | | ns | ns | ns |
| *Dendryphiella* | ns | ns | ns | ns | | ns |  | ns | | ns | ns | ns | ns | |  | * | ns | | ns | * | ns |
| *Diaporthe* | ns | ns | ns | * | | ns |  | ns | | ns | ns | ns | ns | |  | ns | ns | | * | ns | ns |
| *Dioszegia* | ns | ns | ns | ns | | ns |  | * | | ns | * | ns | * | |  | ns | ns | | ns | ns | ns |
| *Emericella* | ns | ns | ** | ** | | * |  | * | | ns | * | ns | * | |  | ns | * | | ** | ** | ** |
| *Epicoccum* | ns | ns | * | * | | * |  | ns | | ns | ** | ns | ns | |  | ns | ns | | ** | * | * |
| *Eurotium* | ns | ns | ns | * | | ns |  | ns | | ns | ** | * | ** | |  | ns | ns | | * | ns | ns |
| *Fennellomyces* | ns | * | ns | * | | * |  | ns | | ns | ns | ns | ns | |  | ns | ns | | ns | ns | ns |
| *Fusarium* | ns | ns | ** | ** | | ** |  | ns | | ns | ** | ns | ns | |  | * | ns | | ** | ** | * |
| *Galactomyces* | ns | ns | ns | ns | | ns |  | ns | | ns | * | ns | * | |  | * | ns | | * | ns | ns |
| *Hypocrea* | ns | ns | * | ns | | ns |  | ns | | ns | ns | ns | ns | |  | ns | ns | | * | ns | ns |
| *Leptosphaeria* | ns | ns | ns | ns | | ns |  | ns | | ns | ns | ns | ns | |  | ns | * | | * | ns | ns |
| *Leucosporidiella* | ns | ns | ns | ns | | ns |  | ns | | ns | ns | ns | ns | |  | ** | ns | | ns | ** | ns |
| *Lewia* | ns | ns | ** | ** | | ** |  | ns | | ns | ** | ns | ns | |  | * | ns | | * | ** | ** |
| *Microdochium* | ns | ns | ns | ns | | ns |  | ns | | ns | ns | ns | ns | |  | ns | * | | * | ns | ns |
| *Monascus* | ns | ns | ** | ** | | ** |  | ns | | ns | ns | ns | ns | |  | ns | * | | ns | ** | * |
| *Monographella* | ns | ns | ns | ns | | ns |  | ns | | ns | ns | ns | ns | |  | ns | ns | | * | ns | ns |
| *Mucor* | ns | ns | * | ** | | * |  | ns | | ns | ns | ns | ns | |  | ns | ns | | * | * | * |
| *Nectria* | ns | ns | * | * | | * |  | ns | | ns | ns | ns | ns | |  | ns | * | | ns | ** | ns |
| *Neocallimastix* | ** | * | ** | ** | | ** |  | ns | | ns | ns | * | ** | |  | ns | ** | | ** | ** | ** |
| *Neosartorya* | ns | ns | ns | ns | | ns |  | ns | | ns | ns | * | ns | |  | ns | ns | | ns | ns | ns |
| *Orpinomyces* | * | ns | ** | ns | | ** |  | * | | ns | ns | ns | ns | |  | ns | ns | | ** | * | ** |
| *Penicillium* | ns | ns | ns | ns | | ns |  | ns | | ns | ns | ns | ns | |  | ** | ns | | ns | ns | * |
| *Phaeosphaeria* | ns | ns | * | ** | | ** |  | ns | | ns | ns | ns | ns | |  | ns | ns | | ** | ns | ns |
| *Phoma* | ns | ns | ** | ** | | ** |  | ns | | ns | * | ns | ns | |  | ns | ns | | ** | ** | ** |
| *Pichia* | ns | * | ** | ** | | ** |  | ** | | ns | ns | ns | ns | |  | ns | ns | | * | ** | ** |
| *Piromyces* | ns | ns | ** | ** | | ** |  | ns | | ns | ns | ns | ** | |  | ns | ns | | * | ** | * |
| *Plectosphaerella* | ns | ns | ** | ns | | ns |  | ns | | ns | ** | ** | ** | |  | * | ns | | ns | * | ns |
| *Pyrenophora* | ns | ns | ns | * | | * |  | ns | | ns | ns | ns | ns | |  | ns | ns | | * | ns | ns |
| *Rhizopus* | ns | ns | * | * | | * |  | ns | | ns | ns | ns | ns | |  | ns | ns | | ns | ** | ns |
| *Rhodotorula* | * | ns | ns | ns | | ns |  | ns | | ns | ns | ns | ns | |  | ns | ns | | ns | ns | ns |
| *Saccharomyces* | ns | ns | ns | ns | | ns |  | ns | | ns | * | * | * | |  | ns | ns | | * | * | ns |
| *Sarocladium* | ns | ns | * | ns | | ns |  | ns | | ns | ns | ns | ns | |  | ns | ns | | ns | * | * |
| *Scedosporium* | ns | ns | * | * | | * |  | ns | | ns | ns | ns | ns | |  | ns | ns | | ** | * | * |
| *Scytalidium* | ns | ns | ** | * | | * |  | ns | | ns | ns | ns | ns | |  | * | ns | | ns | ** | ** |
| *Sporobolomyces* | ns | ns | * | ns | | ns |  | ns | | ns | ns | ns | ns | |  | * | ns | | ns | * | ns |
| *Stagonospora* | ns | ns | ns | ns | | ns |  | ns | | ns | ns | ns | ns | |  | * | ns | | ns | ns | ns |
| *Stemphylium* | ns | ns | * | ns | | * |  | ns | | ns | ns | ns | ns | |  | ns | ns | | * | ns | ns |
| *Thelebolus* | ns | ns | ns | ns | | * |  | ns | | ns | ns | ns | * | |  | ns | ns | | ns | * | ns |
| *Thermomyces* | ns | ns | ns | ns | | ns |  | ns | | ns | ns | ns | ns | |  | * | ** | | ns | * | ** |
| *Trichosporon* | ns | * | ** | ** | | ** |  | ns | | ns | ns | ns | ns | |  | ns | ns | | * | * | ** |
| *Typhula* | ns | ns | ns | ns | | ns |  | ns | | ns | ns | ns | ns | |  | * | ns | | ns | * | ns |
| *Wallemia* | ns | ns | ns | ns | | ns |  | ns | | ns | ns | ns | ns | |  | ns | ns | | ns | * | * |
| *Westerdykella* | ns | ns | ns | * | | ns |  | ns | | ns | ns | ns | ns | |  | ns | ns | | ns | ns | ns |
| *Zymoseptoria* | ns | ns | ns | * | | * |  | ns | | ns | * | * | * | |  | ns | ns | | * | ns | ns |
| unclassified, n | 2 | 3 | 19 | 22 | | 19 |  | 3 | | 0 | 14 | 18 | 16 | |  | 21 | 3 | | 19 | 21 | 13 |

**Supplemental Table 2. Significantly different protozoal species between treatment groups.** Significance is determined as P < 0.05 = * and P < 0.01 ** by Student’s T-Test. Treatments include high-fiber control (HFC), high-fiber yeast (HFY), high-grain control (HGC), and high-grain yeast (HGY). Standard error was < 0.05 for all treatment groups and taxa.

|  | **Epimural** | | | | |  | **Fluid** | | | | |  | **Solid** | | | | |
| --- | --- | --- | --- | --- | --- | --- | --- | --- | --- | --- | --- | --- | --- | --- | --- | --- | --- |
| **taxon** | **HFC x HFY** | **HGC x HGY** | **HFC x HGC** | **HFY x HGY** | **HFC x HGY** |  | **HFC x HFY** | **HGC x HGY** | **HFC x HGC** | **HFY x HGY** | **HFC x HGY** |  | **HFC x HFY** | **HGC x HGY** | **HFC x HGC** | **HFY x HGY** | **HFC x HGY** |
| **Ophryoscolecidae** | | | | | | | | | | | | | | | | | |
| *Diploplastron affine* | ns | ns | ** | * | ns |  | ns | ns | * | ns | ns |  | ns | ns | ns | ns | ns |
| *Entodinium bursa* | ns | ns | * | * | ns |  | ns | ns | * | * | * |  | ns | ns | * | ** | ** |
| *Entodinium caudatum* | ns | ns | * | ** | ** |  | ns | ns | * | ** | * |  | ns | ns | ns | * | * |
| *Entodinium dubardi* | ns | ns | ns | ns | ns |  | ns | ns | * | * | * |  | ns | ns | * | ns | ns |
| *Entodinium furca dilobum* | ns | ns | * | ns | ns |  | ns | ns | * | ns | ns |  | ns | ns | * | ns | ns |
| *Entodinium furca monolobum* | ns | ns | ** | ** | ** |  | ns | ns | ** | ** | ** |  | ns | ns | ** | ** | ** |
| *Entodinium furca* sp. | ns | ns | * | ** | ** |  | * | ns | ns | ns | ** |  | ns | ns | * | ** | ** |
| *Entodinium longinucleatum* | ns | ns | ** | ** | ** |  | ns | ns | ** | ** | ** |  | ns | ns | ** | ** | ** |
| *Entodinium simplex* | ns | ns | ns | * | ** |  | ns | ns | * | ** | * |  | ns | ns | ns | * | ns |
| *Entodinium* sp. | ns | ns | ns | * | ns |  | ns | ns | * | * | ns |  | ns | * | * | ns | ns |
| *Epidinium ecaudatum caudatum* | ns | ns | ns | ns | ns |  | ns | ns | ns | * | ns |  | ns | na | ns | * | ns |
| *Epidinium ecaudatum* sp. | ns | ns | ns | ns | ns |  | ns | ns | ns | ns | ns |  | ns | ns | ns | * | ns |
| *Eremoplastron dilobum* | ns | ns | * | ns | ns |  | ns | na | ns | ns | ns |  | ns | ns | ns | ns | ns |
| *Eremoplastron rostratum* | ns | ns | * | ** | * |  | ns | ns | ** | ** | ** |  | ns | ns | * | ** | ** |
| *Eremoplastron* sp. | ns | * | * | * | * |  | ns | ns | ** | ** | ** |  | ns | ns | * | ** | ** |
| *Eudiplodinium rostratum* | ns | * | ** | ** | * |  | ns | ns | ** | ** | ** |  | ns | ns | * | ** | ** |
| *Eudiplodinium* sp. | ns | na | * | * | * |  | ns | na | * | * | ns |  | ns | na | * | ** | ** |
| *Metadinium medium* | ns | na | ns | * | ns |  | ns | na | ns | * | ns |  | ns | na | * | ns | ns |
| *Metadinium* sp. | ns | na | ns | * | ns |  | ns | na | ns | * | ns |  | ns | na | ns | ns | ns |
| *Ophryoscolex caudatus* | ns | ns | * | ** | * |  | ns | ns | ns | * | * |  | ns | ns | * | ** | ns |
| *Ophryoscolex* sp. | ns | ns | * | ** | * |  | ns | ns | ns | * | ns |  | ns | ns | * | * | ns |
| *Ostracodinium clipeolum* | ns | ns | * | * | * |  | ns | na | na | ns | na |  | ns | ns | ns | ns | ns |
| *Ostracodinium gracile* | ns | * | * | ** | ** |  | ns | ns | * | ns | ** |  | ns | ns | ns | ns | ns |
| *Ostracodinium* sp. | ns | * | ** | ** | ** |  | ns | ns | * | ns | ** |  | ns | ns | * | ** | * |
| *Ostracodinium trivesiculatum* | ns | ns | ** | ** | ** |  | ns | ns | * | ns | ** |  | ns | ns | * | ** | * |
| *Polyplastron* *multivesiculatum* | ns | ns | ** | ** | ** |  | ns | ns | * | * | ** |  | ns | * | ** | ** | ** |
| *Polyplastron* sp. | ns | ns | ns | * | ns |  | ns | ns | ** | ** | ** |  | ns | * | ns | * | ** |
| **Isotrichidae** | | | | | | | | | | | | | | | | | |
| *Dasytricha ruminantium* | ns | ns | ns | ** | * |  | ns | ns | * | ** | * |  | ns | ns | ** | * | ** |
| *Isotricha intestinalis* | * | ns | ns | ns | ns |  | ns | ns | ns | ns | ns |  | ns | ns | ns | ns | ns |
| *Isotricha prostoma* | ns | ns | ns | ns | ns |  | ns | ns | ns | ns | ns |  | ns | ns | ** | * | ** |
| *Isotricha* sp. | ns | ns | ns | ns | ns |  | ns | ns | ns | ns | ns |  | * | ns | ** | * | ** |
| Uncl Entodiniomorphida | ns | ns | ns | ns | ns |  | ns | ns | * | ** | * |  | ns | ns | * | * | ** |
| Uncl Isotrichidae | ns | ns | ns | ** | ns |  | ns | ns | * | ns | * |  | ns | ns | * | ns | ns |
| Uncl Vestibuliferida | ns | ns | ns | * | ns |  | ns | ns | ns | * | * |  | ns | ns | ** | * | ** |
| Uncl Trichostomatia | ns | ns | * | * | ns |  | ns | ns | ns | * | ** |  | ns | ns | ** | * | ** |
| Uncl Litostomatea | ns | ns | ns | ** | ns |  | ns | * | ns | * | * |  | ns | ns | ** | * | ** |
| **Buetschliidae** | | | | | | | | | | | | | | | | | |
| Uncl Buetschliidae | ns | ns | ns | ns | * |  | na | na | na | na | na |  | na | ns | ns | ns | ns |

**Supplemental Table 3. Raw and processed sequence numbers for fungi and protozoal datasets, before normalization.**

|  |  | Fungi | | |  | Protozoa | |
| --- | --- | --- | --- | --- | --- | --- | --- |
| Sample |  | Raw | Processed |  | | Raw | Processed |
| HF_Control_E | Mean | 62,281 | 10,954 | |  | 101,764 | 97,481 |
|  | Min | 24,689 | 648 | |  | 485 | 459 |
|  | Max | 111,380 | 23,409 | |  | 161,266 | 155,709 |
|  |  |  |  | |  |  |  |
| HF_Control_F | Mean | 154,785 | 57,227 | |  | 162,683 | 159,389 |
|  | Min | 10,545 | 1,371 | |  | 104,001 | 103,046 |
|  | Max | 435,515 | 190,819 | |  | 208,880 | 206,963 |
|  |  |  |  | |  |  |  |
| HF_Control_S | Mean | 236,677 | 93,413 | |  | 77,263 | 76,125 |
|  | Min | 58,319 | 9 | |  | 39,076 | 38,677 |
|  | Max | 655,988 | 380,643 | |  | 171,591 | 168,355 |
|  |  |  |  | |  |  |  |
| HF_Yeast_E | Mean | 56,104 | 10,605 | |  | 123,601 | 116,951 |
|  | Min | 24,285 | 4,056 | |  | 53,152 | 51,997 |
|  | Max | 111,797 | 32,179 | |  | 213,846 | 204,152 |
|  |  |  |  | |  |  |  |
| HF_Yeast_F | Mean | 146,607 | 39,631 | |  | 184,615 | 181,383 |
|  | Min | 73,131 | 11,000 | |  | 129,625 | 128,693 |
|  | Max | 242,862 | 101,349 | |  | 358,513 | 354,177 |
|  |  |  |  | |  |  |  |
| HF_Yeast_S | Mean | 136,810 | 31,564 | |  | 75,623 | 74,784 |
|  | Min | 21,296 | 13 | |  | 50,228 | 49,810 |
|  | Max | 262,834 | 77,675 | |  | 120,617 | 119,175 |
|  |  |  |  | |  |  |  |
| HG_Control_E | Mean | 101,667 | 6,182 | |  | 115,029 | 111,019 |
|  | Min | 57,523 | 418 | |  | 84,003 | 82,530 |
|  | Max | 192,006 | 30,143 | |  | 156,394 | 152,217 |
|  |  |  |  | |  |  |  |
| HG_Control_F | Mean | 62,352 | 12 | |  | 82,742 | 82,056 |
|  | Min | 32,121 | 2 | |  | 3,997 | 3,855 |
|  | Max | 100,058 | 26 | |  | 187,987 | 186,619 |
|  |  |  |  | |  |  |  |
| HG_Control_S | Mean | 63,880 | 3,381 | |  | 98,243 | 97,150 |
|  | Min | 18,311 | 511 | |  | 18,886 | 18,559 |
|  | Max | 123,046 | 17,517 | |  | 162,385 | 160,183 |
|  |  |  |  | |  |  |  |
| HG_Yeast_E | Mean | 113,856 | 6,038 | |  | 124,182 | 120,241 |
|  | Min | 53,759 | 302 | |  | 106,886 | 104,516 |
|  | Max | 231,063 | 12,038 | |  | 140,738 | 135,515 |
|  |  |  |  | |  |  |  |
| HG_Yeast_F | Mean | 85,012 | 16 | |  | 97,011 | 96,278 |
|  | Min | 41,325 | 9 | |  | 63,114 | 62,640 |
|  | Max | 148,592 | 31 | |  | 198,012 | 196,523 |
|  |  |  |  | |  |  |  |
| HG_Yeast_S | Mean | 86,646 | 6,616 | |  | 97,949 | 97,088 |
|  | Min | 44,179 | 697 | |  | 36,062 | 35,460 |
|  | Max | 146,089 | 28,610 | |  | 174,769 | 173,230 |
